# Soft-tissue cone-beam computed tomography (ST-CBCT) technique for the analysis of skeletal, dental and periodontal effects of orthopedic rapid maxillary expansion

**DOI:** 10.4317/jced.55139

**Published:** 2018-09-01

**Authors:** Álvaro Furtado, Gisela-Crippa Furtado, Ossam El Haje, Henrique-Damian Rosário, Ademir Franco, Irina Makeeva, Luiz-Renato Paranhos

**Affiliations:** 1 Department of Dentistry, Unifacvest University Center, Brazil; 2 Department of Dentistry, South University of Santa Catarina, Brazil; 3 Department of Therapeutic Stomatology, Institute of Dentistry, Sechenov University, Russia; 4 Department of Preventive and Community Dentistry, School of Dentistry, Federal University of Uberlândia, Brazil

## Abstract

**Background:**

Orthopedic rapid maxillary expansion (RME) is a common treatment of choice for managing transverse deficiency of the maxilla. This approach may have desired and undesired skeletal, dental and periodontal effects that may be assessed clinically or through imaging techniques. This study aims to investigate the dental, skeletal and periodontal effects of orthopedic RME using the soft-tissue cone-beam computed tomography (CBCT) technique.

**Material and Methods:**

The sample consisted of 10 patients (5males and 5 females) aged between 10 and 14 years (mean age: 12.5 years) treated with Hyrax orthopedic device. CBCT scans set for the registration of soft tissue (ST-CBCT) were taken from each patient before (T1) and 120 days after (T2) RME. Skeletal (n=10), dental (n=1) and periodontal (n=4) parameters measured in ST-CBCT were compared between T1 and T2 using t-test within a significance level of 5%.

**Results:**

The skeletal parameters with statistically significant increase (*p*<0.05) in T2 were the width of the buccal alveolar bone crest, the external width of the dental arch at the level of buccal cusps, and the width of the dental arch at the level of most prominent dental surface contour. Representing the dental parameter, the inclination of the anchor teeth was statistically significant for premolars (*p*<0.05). The only statistically significant outcome in periodontal parameters was the decrease in buccal bone plate thickness of first molars (*p*<0.05).

**Conclusions:**

Dentists must be aware of the ST-CBCT technique for the analysis of hard and soft tissue after orthodontic and orthopedic treatments. This technique revealed that the RME reached optimal skeletal and dental effects with minimal periodontal side effects.

** Key words:**Cone-beam computed tomography, imaging, orthodontics, orthopedics.

## Introduction

The transverse deficiency of the maxilla (TDM) may lead to posterior crossbite and must be treated with specific and optimal therapeutic approaches. The rapid maxillary expansion (RME) figures as a clinical tool for the treatment of TDM in Orthodontics (1,2) The orthopedic devices used in the RME promote opening of the median palatal suture (3-6); buccal positioning of the anchor teeth with inclination and translation (4,7); central positioning of the mandibular condyles in the glenoid fossa and major asymmetry between them (1); increase in the width of the nasal cavity (4,6,7); slight positioning of the maxilla downwards and extrusion of the upper molars (8); and buccal inclination of the crowns of the supporting teeth with reduction of the buccal bone plate thickness (9).

The soft-tissue cone-beam computed tomography (ST-CBCT) (10,11) technique emerged in the last decade to enable more accurate analyses of the periodontal structures, such as the thickness of palatal mucosa and gingiva. In this technique, conventional CBCT scans are taken while the patient uses a lip retractor to expose intraoral tissues for a better image acquisition (10,11). Despite the vast scientific literature on the RME, no study was designed to investigate the skeletal, dental and periodontal effects of RME through the ST-CBCT technique. In this context, this study innovates in the interface of Imaging and Orthodontics and contributes towards optimal and evidence-based clinical practices.

Based on the exposed, the present study aims to use the ST-CBCT technique to exam the skeletal, dental and periodontal effects of RME in patients that underwent treatment for TDM.

## Material and Methods

This study was approved by the local Committee of Ethics in Research under the protocol number 173/11.

A longitudinal experimental study was designed, in which the patients were selected based on their registration for orthodontic treatment at university level.

Inclusion criteria were used to filter an initial sample of 420 patients. These criteria consisted of selecting male and female patients aged between 10 and 14 years old (mean age: 12.5 years). After the selection process, exclusion criteria were applied to the remaining 157 patients. According to these criteria, patients with facial profile type III were excluded from the sample, as well those with Angle’s occlusal relation other than Class I or II division 1. Additionally, patients with overbite below -1mm and above +4mm were excluded. Other exclusion criteria consisted of deciduous molars in the maxillary arch, metallic restoration in the permanent maxillary first molars, periodontal disease and history of previous orthodontic treatment. After exclusions, the sample was reduced to 82 patients. Next, an experienced orthodontist examined each patient in the search for those with maxillary atresia (considering the clinical aspect of the buccal corridor). Out of the selection process 21 patients were considered eligible for RME. In the next phases 2 patients quit the treatment and 9 needed new activations (prolonged treatment). Based on that, they were excluded from the sample. The final sample remained with 10 patients.

Before the treatment with RME each patient underwent CBCT scan for optimal orthodontic planning. The images were taken with the iCat Vision (Imaging Sciences International, Hatfield, PA, USA) device set with 120kVP, 37mAs and acquisition time 14.7 seconds. During image acquisition the ST-CBCT technique was used (10,11). This technique consists of positioning an orthodontic lip retractor to enable a clearer view of the soft periodontal tissue (10,11). For a better analysis of the maxillary teeth, the images were not taken with the teeth in occlusion. All the images were taken by a single technician in the same CBCT device.

RME was performed with Hyrax orthopedic device supported bilaterally in the maxillary first premolars and molars. An expansion screw (Morelli, Sorocaba, SP, Brazil) of 13mm was used and the activation was performed (full rotation) right after the installation of the device and daily with 1⁄4 rotation in the morning and 1⁄4 in the evening (4,7). RME was concluded when the palatal cusps of the maxillary first premolars reached contact with the buccal cusps of the mandibular first premolars (12). Clinical follow-ups were performed weekly until the treatment was finished. After RME, the activation screw was locked for 120 days. During this period, the clinical follow-up was conducted once a month. After 120 days, new ST-CBCT scans were taken. The ST-CBCT data before (T1) and after (T2) RME were reconstructed from DICOM files in 0.5mm increments. Image analysis was performed with OsiriX v.5.5.2 (Osirix Foundation, Bernex, Switzerland) software package. The maxilla was examined in axial, sagittal and coronal slices.

**Figure 1:**
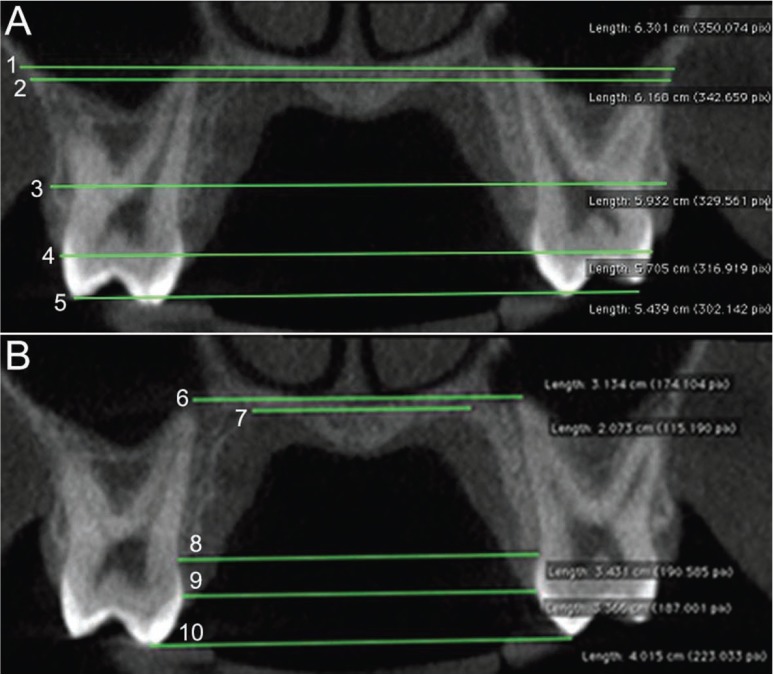
External (A) and internal (B) measurements (n=10) taken through ST-CBCT in each patient to analyze the skeletal effects of rapid maxillary expansion. Legend: Measurements from 1 to 10 correspond, respectively, to the skeletal parameters NF, HP, BAC, DA.E’, DA.E, HP’, PA, LAC, DA.I’ and DA.I. Additional description of these parameters is found in  Table 1.

**Figure 2:**
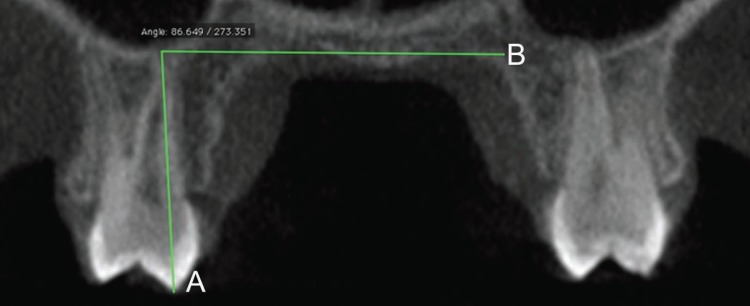
Angle measured between a line from the palatal cusp tip to apex of the anchor tooth (A) and a horizontal line the at the level of hard palate (B). Legend: This measurement expressed to the angle of anchor tooth quantified through ST-CBCT in each patient before and after rapid maxillary expansion.

**Figure 3:**
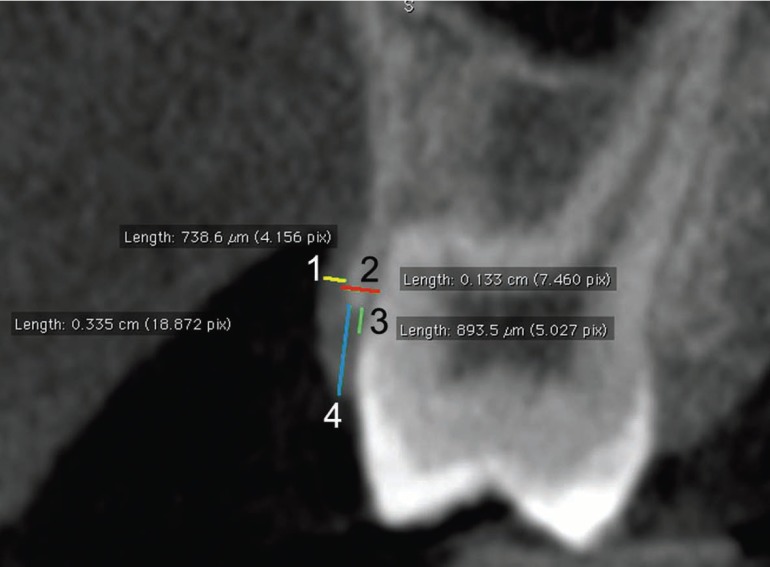
Measurements (n=4) taken through ST-CBCT in each patient to analyze the periodontal effects of rapid maxillary expansion. Legend: Measurements from 1 to 4 correspond, respectively, to the periodontal parameters TAG, BBPT, CJABC and GMABC. Additional description of these parameters is found in  Table 1.

 Table 1 describes the skeletal (n=10; Fig. 1), dental (n=1; Fig. 2) and periodontal (n=4; Fig. 3) parameters measured in each patient through ST-CBCT in T1 and T2 ( Table 1). The systematic error intra-examiner was assessed with paired t-test, while Dahlberg test (error=√∑d2/2n, in which d: difference between the 1st and the 2nd measurements and n: number of repetitions) was used to assess the casual error (13). All the measurements were taken twice within an interval of 15 days. The difference between measurements taken in T1 and T2 was explored in descriptive statistics and compared using paired t-test and considering a significance level of 5% (*p*<0.05). All the statistical procedures were performed with Statistica 11 (StatSoft Inc., Tulsa, USA) software package.

**Table 1:**
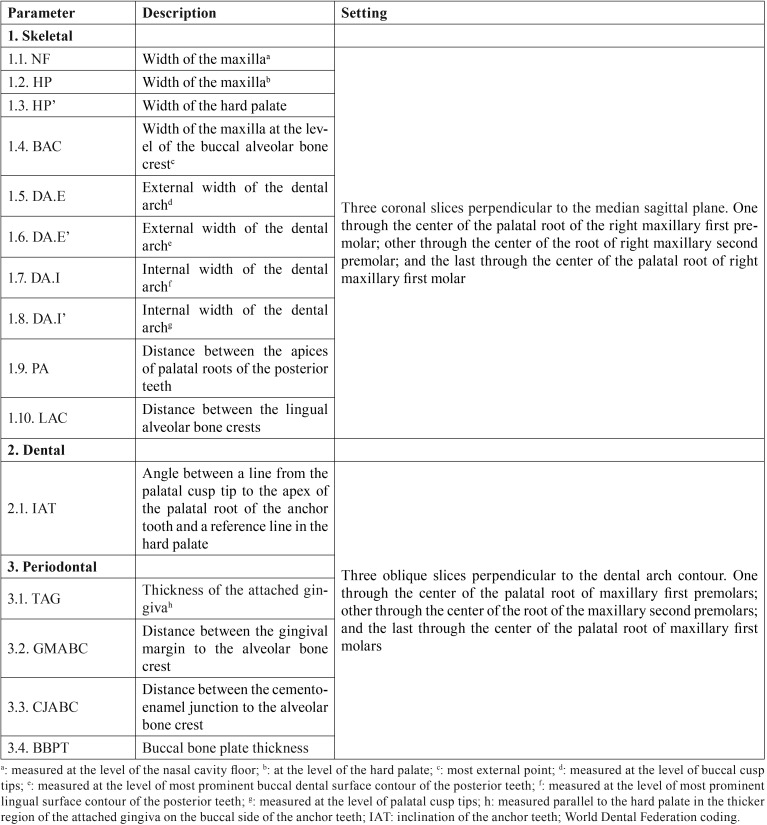
Skeletal, dental and periodontal effects of the rapid maxillary expansion analyzed through soft-tissue cone-beam computed tomography (ST-CBCT).

## Results

Paired t-test and Dahlberg test demonstrated that the systematic and casual errors intra-examiner resulted without statistically significant differences (p>0.05) between the two measurements performed by the same examiner. This outcome was observed for the measurements of all parameters (skeletal, dental and periodontal).

The mean and standard deviations of the skeletal measurements in T1 and T2 are reported in  Table 2. Statistically significant increase in size was observed in the width of the maxilla at the level of buccal alveolar bone crest in the region of first premolars and molars; in the external width of the dental arch at the level of buccal cusp tips; and in the external width of the dental arch at the level of the most prominent buccal dental surface contour of the posterior teeth (*p*<0.05).

**Table 2:**
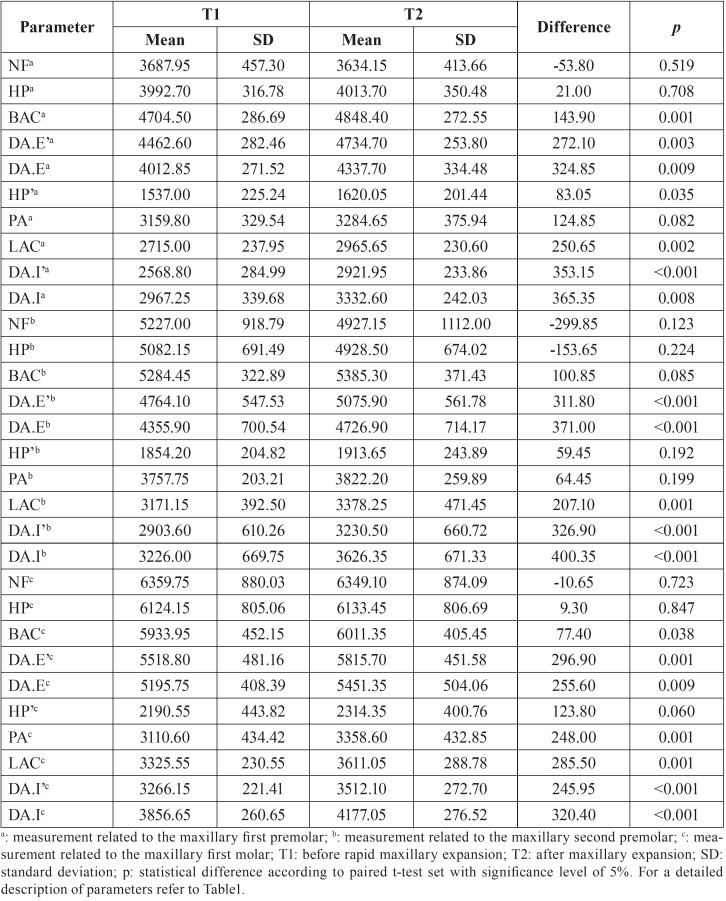
Comparison of skeletal parameters measured in each patient before and after rapid maxillary expansion.

The mean and standard deviations of the measurements taken from the inclination of the anchor teeth in T1 and T2 are reported in  Table 3. The only statistically significant difference was observed in the premolars, which increased their inclination (angle) in T2 (*p*<0.05).

**Table 3:**
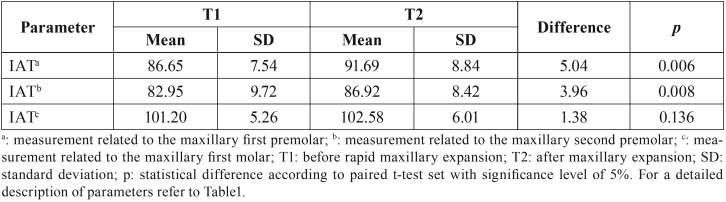
Comparison of dental parameters measured in each patient before and after rapid maxillary expansion.

The mean and standard deviations of the measurements taken from the condition of the periodontal structures of the anchor teeth before (T1) and after (T2) RME are reported in  Table 4. A general decrease in measurements was observed after RME. Statistically significant decrease in the buccal bone plate thickness was observed in the molar region (*p*<0.05). The other periodontal structures differed between T1 and T2 without statistical significance (*p*>0.05).

**Table 4:**
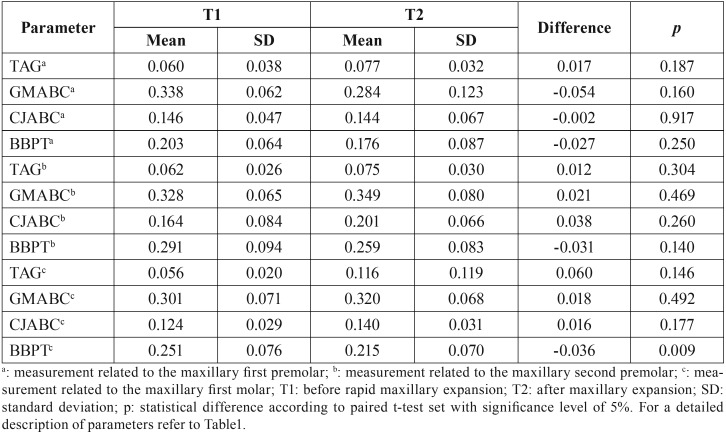
Comparison of periodontal parameters measured in each patient before and after rapid maxillary expansion.

## Discussion

Recent studies (14-17) used (CB)CT to investigate the effects of orthodontic and orthopedic treatments in dental and maxillofacial structures. This type of imaging modality enables a highly accurate access to human hard tissues. However, according to Januário *et al.* (10) (2008), this exam also could play a valuable role for the analysis of periodontal soft tissues. The present study used the CBCT technology to perform skeletal, dental and periodontal measurements (10,11) in patients that underwent RME. Moreover, this study innovates by investigating also alterations in periodontal soft tissue after RME.

Initially, this study did not reveal a statistically significant increase in the transverse dimension of the maxilla at the level of nasal cavity floor and external hard palate. Differently, other authors observed significant effects of RME in these structures (4,7,14,16,17). In practice, the difference may be justified in the inherent characteristics of the sample and in the protocol used for the activation of the orthopedic device. The patients sampled in this study had maxillary atresia but did not have posterior crossbite. It justifies the need for a minor expansion compared to other studies. For this reason, an exact quantity of activation and expansion was not established beforehand. Following Christie *et al.* (6) (2010) and Pangrazio-Kulbersh *et al.* (16) (2012), clinical parameters were adopted to indicate the necessary quantity of expansion in each patient. Consequently, the opening of the median palatal suture observed in T2 promoted a significant increase in size in the internal measurements of the hard palate (at the premolar region) but not in the external measurements. These outcomes suggest that the RME had discrete skeletal effects in the basal structures of the maxilla, such as the hard palate.

Other measurements that revealed statistically significant increase in size were the width of the maxilla at the level of buccal alveolar bone crests and the external width of the dental arch. The previous scientific literature (3,4,7,14,16,17) corroborates these outcomes and shows that the RME triggers not only orthopedic but also orthodontic effects by remodeling the alveolar bone. This outcome confirms the previous findings and indicates that the RME led to major modifications in the alveolar bone compared to the hard palate (7,14). This phenomenon potentially relies on the advanced stage of bone formation given by the age of the patients and also by the lack of deciduous teeth.

Other orthodontic effects reported in the scientific literature (3,9,7,14,17) is the buccal inclination of the anchor teeth. These effects were also detected in this study. However, according to the literature (7,9,14) the second premolars are more susceptible to inclination because usually they are not banded. Differently, both the first and second premolars had a significant inclination in the dental arch.

The buccal bone plate thickness of premolars and molars decreased after RME. However, statistically significant differences between T1 and T2 were observed only in molars. Studies in the field already detected these alterations (7,9,14). Rungcharassaeng *et al.* (9) (2007) observed more evident decrease in the measurement of thickness in the buccal bone plate of first molars, followed by first premolars and second molars. Moreover, these outcomes show that teeth with orthodontic bands and under direct expansion force are more susceptible to adverse effects. Oppositely, the present study demonstrated a gradual decrease from the first premolar (less evident) to the first molar (more evident and statistically significant).

The buccal gingival thickness of the anchor teeth was not altered significantly 120 days after RME. Artun and Grobéty (18) (2001) observed that the significant inclination of a tooth may be performed without risk of gingival retraction. However, this type of orthodontic movement may lead to bone dehiscence. This adverse effect may be more justified in the decrease in the buccal bone plate thickness than in the decrease in the thickness of attached gingiva. Additionally, other factors, such as the dental plaque control and the lack of trauma by tooth brushing, are essential to maintain proper conditions of periodontal structures (19). In the same context, Greenbaum and Zachrisson (20) (1982), followed during 3 years a group of patients that underwent RME and another that were not treated with RME. The authors observed proper periodontal condition in both groups. However, most of the patients with insertion loss in the buccal side of molars were treated with RME. On the other hand, the study also highlights that individual variations were determinant to the occurrence of adverse periodontal effects. The outcomes of the present study show that despite the decrease in the buccal bone plate thickness, bone dehiscence was not detected – which is a positive evidence for periodontal maintenance.

The benefits of the RME are well established in the scientific literature. In parallel, high-tech imaging and techniques, such as CBCT and ST-CBCT, respectively, point towards more predictable adverse effects. The present study showed that the skeletal, dental and periodontal effects of RME are evident. Additionally, it shows that in short-term and non-excessive treatment the buccal gingival tissue is not significantly altered by the RME. However, this study recommends further investigations to verify the buccal gingival tissue of patients that undergo RME with the need for larger expansion.
